# Altered gyrification in chemotherapy-treated older long-term breast cancer survivors

**DOI:** 10.21203/rs.3.rs-2697378/v1

**Published:** 2023-04-10

**Authors:** Ebenezer Daniel, Frank Deng, Sunita K. Patel, Mina S. Sedrak, Heeyoung Kim, Marianne Razavi, Can-Lan Sun, James C. Root, Tim A. Ahles, William Dale, Bihong T. Chen

**Affiliations:** City of Hope National Medical Center: City of Hope; City of Hope National Medical Center: City of Hope; City of Hope National Medical Center; City of Hope National Medical Center: City of Hope; City of Hope National Medical Center: City of Hope; City of Hope National Medical Center; City of Hope National Medical Center; Memorial Sloan Kettering Cancer Center; Memorial Sloan Kettering Cancer Center; Memorial Sloan Kettering Cancer Center; City of Hope National Medical Center

**Keywords:** Gyrification index, Breast cancer, Cancer-related cognitive impairment, Chemotherapy

## Abstract

**Purpose:**

The purpose of this prospective longitudinal study was to evaluate the changes in brain surface gyrification in older long-term breast cancer survivors 5 to 15 years after chemotherapy treatment.

**Methods:**

Older breast cancer survivors aged ≥ 65 years treated with chemotherapy (C+) or without chemotherapy (C−) 5–15 years prior and age & sex-matched healthy controls (HC) were recruited (time point 1 (TP1)) and followed up for 2 years (time point 2 (TP2)). Study assessments for both time points included neuropsychological (NP) testing with the NIH Toolbox cognition battery and cortical gyrification analysis based on brain MRI.

**Results:**

The study cohort with data for both TP1 and TP2 consisted of the following: 10 participants for the C+ group, 12 participants for the C− group, and 13 participants for the HC group. The C+ group had increased gyrification in 6 local gyrus regions including the right fusiform, paracentral, precuneus, superior, middle temporal gyri and left pars opercularis gyrus, and it had decreased gyrification in 2 local gyrus regions from TP1 to TP2 (*p* < 0.05, Bonferroni corrected). The C− and HC groups showed decreased gyrification only (*p* < 0.05, Bonferroni corrected). In C+ group, changes in right paracentral gyrification and crystalized composite scores were negatively correlated (*R* = −0.76, p = 0.01).

**Conclusions:**

Altered gyrification could be the neural correlate of cognitive changes in older chemotherapy-treated long-term breast cancer survivors.

## Introduction

More than 4 million women have a history of breast cancer, and additional newly identified 287,850 cases have been reported as of January 1, 2022 in the United States alone [1]. Besides, more than 2.7 million breast cancer survivors are 65 years and older [1]. Prior studies have shown that chemotherapy-treated breast cancer survivors suffer from cancer-related cognitive impairment (CRCI) [2-4]. CRCI mainly affects memory, attention, and executive functioning in older long-term survivors [5-7].

Neuroimaging studies have shed light on brain structural and functional alterations underlying CRCI in breast cancer survivors [8-10]. Previous studies have found a significant reduction in brain gray matter (GM) and white matter in long-term breast cancer survivors at ten or even twenty years after chemotherapy [11-13]. GM atrophy has been known to have a significant association with cognitive dysfunction amongst breast cancer survivors [14-20]. Our previous study of older breast cancer survivors showed cortical thinning in older long-term breast cancer survivors [9].

Cortical Gyrification is a morphometric feature related to the geometry of the brain surface [21, 22]. Since GM forms an outer layer of the brain surface, the alterations in gyrification result in changes in cortical surface area and cortical GM volume [23]. Gyrification analysis focuses on brain morphometric features that are not identified by GM or cortical thickness [24]. During brain development, gyrification increases and peaks during childhood, promptly decreases during the adolescent stage and then gradually decreases with age [24-27]. Thus, gyrification is expected to decrease with aging [24]. The decreased gyrification is considered an early morphometric biomarker for cognitive changes in patients with Alzheimer’s disease (AD) [28], subjective cognitive impairment [29], autism [30], mild traumatic brain injury [31] and in healthy individuals with normal aging [32]. In addition to the decreased gyrification patterns, prior studies on schizophrenia [24], AD [33], traumatic brain injury [34] and autism [35] have also showed increased gyrification patterns which was associated with cognitive impairment. A previous study of CRCI showed decreased gyrification in patients with breast cancer aged 29 to 68 years shortly after chemotherapy [36]. However, there is limited literature on gyrification in older long-term breast cancer survivors.

Here, we conducted a longitudinal study to assess the brain surface gyrification changes in older breast cancer survivors. We hypothesized that gyrification would be decreased in the older long-term breast cancer survivors with exposure to chemotherapy, which would be correlated with cognitive changes. To test this hypothesis, we assessed brain gyrification on brain MRI and cognitive performance via neuropsychological (NP) testing in older breast cancer survivors who had chemotherapy treatment 5–15 years prior to enrollment and compared this group to the two control groups including the no-chemotherapy group and healthy control group over two years.

## Methods

### a. Subjects

The study was a neuroimaging sub-study of a multicenter trial of long-term breast cancer survivors (parent trial: Cognition in Older Breast Cancer Survivors: Treatment Exposure, APOE and Smoking History, NCT02122107). Breast cancer survivors treated with chemotherapy (C+) or without chemotherapy (C−) 5-15 years prior and age & sex-matched healthy controls (HC) with no history of cancer were enrolled. All participants were aged ≥ 65 years at the time of initial enrollment. Study assessment included brain MRI and NP attesting with the National Institute of Health (NIH) Toolbox Cognition Battery both at time point 1 (TP1) upon enrollment and at the 2-year interval at time point 2 (TP2). The eligibility criteria for breast cancer survivors were the following: woman aged 65 years and older with a history of stage I-III breast cancer with or without chemotherapy treatment at 5 to 15 years after surviving breast cancer, and no contraindications such as orbital metal or claustrophobia for brain MRI scans. Exclusion criteria included the following: history of stroke, psychiatric disease, metastatic disease, or any other cancer. Age and sex-matched HCs were enrolled with similar criteria except for the history of cancer. The HCs were recruited via local newspaper advertisements, patient referrals, and community health fairs. This study was approved by the Institutional Review Board (IRB) of City of Hope National Medical Center. Written informed consent was obtained from all participants in compliance with institutional guidelines and the Declaration of Helsinki, as well as local, state, and federal regulations from all participating subjects.

### b. MRI acquisition and gyrification analysis

All brain MRI scans were acquired for both TP1 and TP2 in the same in-house 3T VERIO Siemens scanner (Siemens, Erlangen, Germany). Structural three-dimensional (3D) T1-weighted magnetization prepared rapid gradient echo (MPRAGE) images were acquired with the following parameters: TR = 1900 millisecond (ms), TE = 2.94 ms, inversion time = 900 ms, FA = 9°, and voxel size = 0.45 x 0.45 x 1.5. Incidental brain pathology was assessed on the T1-weighted MPRAGE and fluid-attenuated inversion recovery (FLAIR) images by the neuroradiologist in the study (BC). The cortical gyrification analysis was performed using the Computational Anatomy Toolbox (CAT12) [24] from the T1-weighted images. All images were manually re-oriented using the statistical parametric mapping toolbox (version SPM12) (Wellcome Department of Cognitive Neurology, UK). The CAT12 and SPM12 toolboxes for our analysis were based on MATLAB (R2019b). The mean gyrification values were analyzed based on Desikan-Killiany (DK40) cortical atlas [37, 38]. We followed the standard pipeline and settings for preprocessing and gyrification analysis [24]. The main steps were as follows: i) extraction of central surface, ii) estimation of the local absolute mean curvature from each vertex point within the 3 mm of this central surface given point, iii) smoothing and resampling of the gyrification maps using full width at half maximum (FWHM) gaussian filter at 20 mm.

### c. NP testing with NIH toolbox for cognition

The NP testing was performed using the NIH Toolbox Cognition Battery [39, 40]. This cognitive testing battery generated seven individual scores for List Sorting Working Memory, Picture Sequence Memory, Pattern Comparison Processing Speed, Oral Reading Recognition, Picture Vocabulary, Flanker Inhibitory Control, and Dimensional Change Card Sorting. Additionally, the crystalized, fluid, and total composite cognition scores, were also generated.

### d. Statistical analysis

Clinical and demographic information was assessed using analysis of variance (ANOVA) for continuous variables. Categorical variables were analyzed using Fisher’s exact tests. Threshold of *p*-value at 0.05 was considered statistically significant for both continuous and categorical variables, and all tests were two-sided.

NP test performance was analyzed using a generalized linear model (GLM) with the correlation of repeated measurements within subjects [9, 41]. Group (C+, C−, HC) and time-point (TP1, TP2) were considered categorical fixed effects in this analysis. Using the GLM, we tested the following: 1) whether there were any differences in NP scores between the 3 groups at TP1 or TP2, 2) whether there were any significant longitudinal differences within group, 3) whether there was a group by time interaction effect. SAS 9.3 (SAS Institute, Cary, NC) was used for data analyses.

Whole brain surface gyrification was compared between groups at TP1 using two-sample t-test. Within group longitudinal change over the 2-year study interval was tested using paired t-tests. In both analyses, effects were corrected for multiple comparisons for the whole brain using Bonferroni correction in the CAT12 software with a significance threshold of *p* < 0.05. The correlations of the mean gyrification values with NP composite scores were tested using linear regression analysis with a *p-value* of 0.05 being considered significant. The linear regression analysis and group by time interaction were tested using the statistical package for the social science software (SPSS, v 27, Chicago, IL).

## Results

### a. Demographic data

At TP1, a total of 60 participants were enrolled with 20 participants for each of the three groups, i.e., C+, C− and HC groups. At TP2, due to attrition from loss to follow-up, new cancer, new memory problems, refusal and death, the cohort consisted of 10 participants for the C+ group, 12 participants for the C− group, and 13 participants for HC group [9]. There were no significant differences among the groups in age (*p* = 0.75), education (*p* = 0.80) or race (*p* = 0.37) (Table 1). More detailed clinical and demographic information for this cohort has been reported [9]. In the C+ group, 80% of survivors had Stage II breast cancer. The C− group consisted of 50% survivors in stage 0, 33% survivors in stage I and 17 *%* survivors in stage II. In the C+ group, 90% of survivors had treatment with non-trastuzumab regimen and 10% of survivors with trastuzumab regimen (Table 1).

### b. Gyrification results

There were no significant gyrification differences at TP1 between C+ versus C−, C+ versus HC, and C− versus HC (*p* > 0.05, Bonferroni corrected).

**Figure 1:**
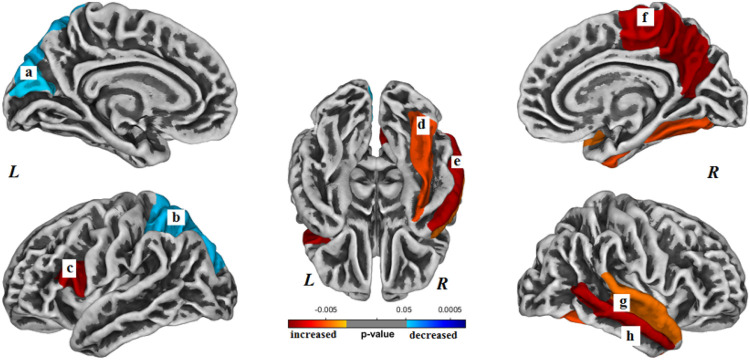
Brain regions with longitudinal changes in gyrification within the chemotherapy (C+) group. The altered regions are (a) left cuneus, (b) left superior parietal gyrus, (c) left pars opercularis, (d) right fusiform gyrus, (e) right middle temporal gyrus, (f) right precuneus, (g) right superior temporal gyrus, and (h) right middle temporal gyrus. *L*- left hemisphere, *R*- right hemisphere. Results were Bonferroni correctedat significant level of 0.05.

Within the C+ group, gyrification was significantly increased in 6 regions and decreased in 2 regions longitudinally over the 2-year study interval (*p* < 0.05, Bonferroni corrected) (Table 2). The brain regions with increased surface gyrification in the C+ group included the following (Bonferroni corrected): left pars opercularis gyrus (*p* < 0.001), right superior temporal gyrus (*p* < 0.001), right middle temporal gyrus (*p* < 0.001), right precuneus gyrus (*p* < 0.001), right paracentral gyrus (*p* < 0.001) and right fusiform gyrus (*p* = 0.004) (Fig. 1). The brain regions with decreased surface gyrification in the C+ group included the following (Bonferroni corrected): left superior parietal gyrus (*p* = 0.030) and left cuneus gyrus (*p* = 0.030).

**Figure 2:**
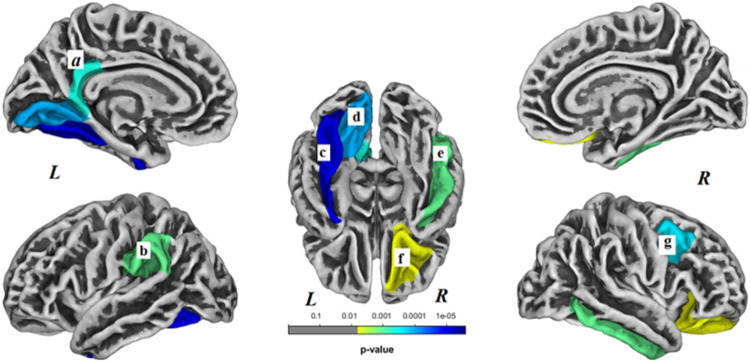
Brain regions with decreased gyrification within the non-chemotherapy control (C−) group. These regions included the following: (a) left isthmus cingulate gyrus, (b) left supramarginal gyrus, (c) left fusiform gyrus, (d) left lingual gyrus, (e) right inferior temporal gyrus, (f) right lateral orbitofrontal gyrus, (g) right caudal middle frontal gyrus. *L*- left hemisphere, *R*- right hemisphere. Results were Bonferroni corrected at significant level of 0.05.

Within the C− group, brain surface gyrification was significantly decreased in 7 regions (*p* < 0.05, Bonferroni corrected) and no regions showed increased gyrification. Decreased surface gyrification within the C− group was noted in left fusiform gyrus (*p* < 0.001), left lingual gyrus (*p* < 0.001), left isthmus cingulate gyrus (*p* < 0.001), left supramarginal gyrus (*p* = 0.001), right lateral orbitofrontal gyrus (*p* = 0.006), right inferior temporal gyrus (*p* = 0.001) and right caudal middle frontal gyrus (*p* < 0.001) (Fig. 2).

**Figure 3:**
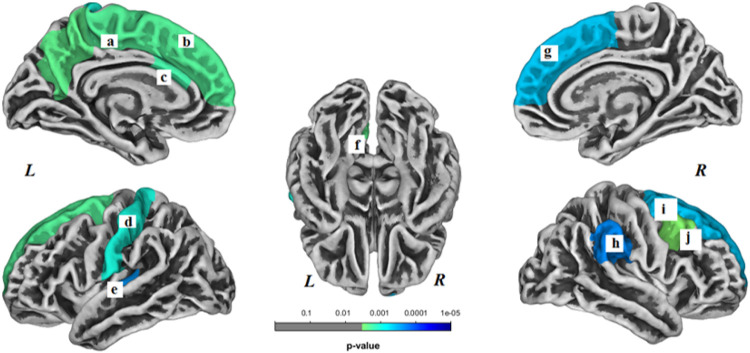
Brain regions with decreased gyrification within the healthy control (HC) group. These regions included the following: (a) left paracentral gyrus, (b) left superior frontal gyrus, (c) left caudal anterior cingulate gyrus, (d) left postcentral gyrus, (e) left transverse temporal gyrus, (f) left precuneus, (g) right superior frontal gyrus, (h) right supramarginal gyrus, (i) right superior frontal gyrus, and (j) right caudal middle frontal gyrus. *L*- left hemisphere, *R*- right hemisphere. Results were Bonferroni correctedat significant level of 0.05.

In the HC group, brain surface gyrification was significantly decreased in 9 regions (*p* < 0.05, Bonferroni corrected) and no regions showed increased gyrification longitudinally. Decreased brain surface gyrification was noted in the following regions: left superior frontal gyrus (*p* < 0.001), left postcentral gyrus (*p* < 0.001), left precuneus gyrus (*p* < 0.001), left paracentral gyrus (*p* < 0.001), left caudal anterior cingulate gyrus (*p* < 0.001), left transverse temporal gyrus (*p* < 0.001), right superior frontal gyrus (*p* < 0.001), right caudal middle frontal gyrus (*p* < 0.001) and right supramarginal gyrus (*p* < 0.001) (Fig. 3).

There was no significant gyrification difference noted in group-by-time interaction analysis (*p* > 0.05, Bonferroni corrected).

### c. NP testing scores

The detailed results of the NIH Toolbox cognition battery testing scores have been reported in our prior study of cortical thickness in the same cohort [9]. Briefly, the C+ group showed significantly decreased total composite score (*p* = 0.01), fluid composite score (*p* = 0.03) and picture vocabulary score (*p* = 0.04) across the 2-year interval. No significant changes in NP scores were noted in C− and HC group at a threshold of *p* values at 0.05.

### d. Correlation between gyrification and NP scores

**Figure 4:**
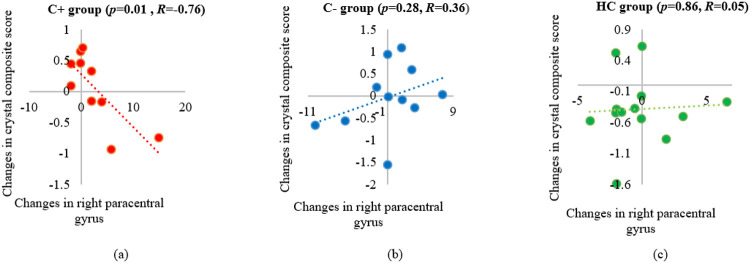
Correlation of longitudinal changes between the right paracentral gyrification values and the crystallized composite scores. (a) chemotherapy (C+) group, (b) no-chemotherapy (C−) group, and (c) healthy control (HC) group. *R*: the Pearson’s correlation coefficient with significance set at *p* ≤ 0.05.

The correlation analysis was performed between the significant gyrification alterations within each group over time and the 3 NP composite scores. A significant negative correlation was noted between longitudinal changes in the crystallized composite scores and right paracentral gyrification values in the C+ group (*p* = 0.01, *R* = −0.76). No significant correlations were noted in the C− or the HC group (Fig. 4).

## Discussion

We identified altered gyrification in the older long-term survivors of breast cancer with exposure to chemotherapy. We found the mostly increased gyrification in the chemotherapy-treated group while only decreased gyrification in the control groups over the 2-year study interval. In addition, we also found a significant correlation between the increased gyrification and the changes in cognitive testing scores. To the best of our knowledge, this was the first prospective longitudinal study of the effect of chemotherapy on gyrification in older long-term survivors of breast cancer.

Our C+ group showed increased gyrification in the right superior temporal gyrus. In contrast, a previous study of breast cancer patients with neoadjuvant chemotherapy showed decreased gyrification in the same region [36]. The divergent results might be due to differences in study designs. The prior study focused on the acute effects of chemotherapy within 2 months after treatment and assessed the pre- and post-chemotherapy differences in patients of 29 to 68 years of age [36]. Therefore, this prior study assessed acute changes stimulated by neurotoxic effects of chemotherapy while our study assessed the chronic chemotherapy-related neurotoxicity in older breast cancer survivors. Literature supports this pattern of gyrification alteration with decreased gyrification in acute phase as noted in mild traumatic brain injury within 3 months of brain injury [31] and increased gyrification in a cohort with childhood traumatic brain injury after 6 to 15 years of post-injury [34]. Brain changes associated with chemotherapy tend to be subtle and are similar to mild traumatic brain injury. One speculation for the increased gyrification relies on the phenomena of neurogenesis [33], in which the brain might expand by increasing gyrification to accommodate newly generated neurons. In addition, the right superior temporal gyrus plays a role in social cognitive function such as auditory and language processing [42]. The oral reading recognition score from the NP testing in our study assessed language and auditory skills [43] and was decreased within the C+ group, thus implying the brain structure including the superior temporal gyrus underlying these functions, may be altered. Therefore, we speculate that the increased right superior temporal gyrification might be a compensatory measure to accommodate the newly generated neurons to counter neurotoxicity of chemotherapy [33].

We found increased gyrification in the right medial temporal gyrus in the C+ group, which is in general agreement with a prior study in patients with early stages of dementia [33]. Patients with mild cognitive impairment (MCI) and Alzheimer’s Dementia (AD) [33] had increased gyrification and atrophy in entorhinal cortex, which is a part of the medial temporal gyrus. The medial temporal lobe is associated with episodic memory [44]. We also found a decreased picture vocabulary testing score in the C+ group, indicating diminished episodic memory. Our findings support the notion that gyrification alteration in the medial temporal lobe may be useful as an imaging biomarker for CRCI and AD in older cancer survivors. The right fusiform gyrus, close to the medial temporal gyrus, also showed increased gyrification in our C+ group. The fusiform gyrus plays an important role in semantic memory such as face recognition [45], visual perception [46] and face stimuli [46]. Our own prior study noted GM reduction in the right fusiform cortex in the chemotherapy-treated group [47]. Overall, our findings implicate the temporal lobe structures as being vulnerable to chemotherapy neurotoxicity.

We found increased gyrification in the paracentral gyrus within the C+ group over time and our findings were consistent with a prior study showing decreased sulcus depth in the paracentral gyrus during the early post-chemotherapy phase in breast cancer patients [36]. The paracentral gyrus is the medial continuation of the precentral and postcentral regions, which controls motor and sensory innervations of the contralateral lower extremity [48]. Our findings implicate the paracentral gyrification as a potential neural correlate for CRCI in older long-term cancer survivors who had chemotherapy treatment many years ago. The increased gyrification in the paracentral gyrus region had a significant negative association with the crystallized composite scores in the C+ group. The crystalized intelligence consisted of picture vocabulary and oral reading recognition based on past learning experiences [49]. Nevertheless, the crystalized cognition score was only marginally significant overtime in our C+ group and this score has been known to be resilient to change [50]. More studies in larger samples are needed to confirm the association of crystalized composite score and paracentral gyrification changes in the older survivors treated with chemotherapy.

We found decreased gyrification in the left superior parietal lobe in the older long-term breast cancer survivors with history of chemotherapy treatment. A prior study showed similar findings in a cohort of breast cancer patients shortly after chemotherapy [36]. The parietal lobe is important for cognitive function, and atrophy of the superior partial lobe is associated with impairment of working memory, attention and visuomotor functions [51, 52]. Taken together, the diminished left superior parietal gyrification may have occurred shortly after chemotherapy and persisted into long-term survivorship. Nevertheless, a longitudinal study including a pre-chemotherapy baseline and long-term follow-up is needed to assess the trajectory of gyrification alterations.

The control groups in our study showed only decreased gyrification over time with no increase noted, which was consistent with prior studies of normal aging. For instance, a prior study has shown decreased gyrification in the older population as compared to the younger population [32]. The decreased gyrification in the left lingual and right lateral orbitofrontal gyrus in our C− group and in the left postcentral and precuneus in the HC group were in line with previous longitudinal study of healthy aging [33]. The underlying neural mechanism for decreased gyrification in the aging studies is not well known [24]. We speculate that it could be partly due to age-related brain volume loss, leading to less folding of gyrus thus decreased gyrification during the aging process [24].

There were limitations to this study. First, our study cohort was small and there was severe attrition during the 2-year study interval. We will implement measurements and lessons from this study to decrease attrition in our future studies. Second, our study cohort included mostly non-Hispanic white women, decreasing the generalizability of our gyrification results to other racial and ethnic groups. Third, though gyrification is a significant surface parameter to assess brain alterations, other surface morphology parameters such as sulcal depth may help confirm brain changes. Further analysis of brain surface parameters is ongoing. Lastly, we only identified longitudinal changes over a 2-year interval but not at TP 1 during the initial enrollment. We believe a larger sample size may have detected subtle differences among the groups at TP 1. Despite the limitations, this study has merits. This was the first longitudinal study to assess the effect of chemotherapy on gyrification in older long-term survivors of breast cancer. We contributed novel brain structural and functional information to advance CRCI research in older cancer survivors.

## Conclusions

We identified altered brain surface gyrification and its association with cognitive function in long-term breast cancer survivors who had chemotherapy many years ago. This study implicated gyrification as a possible underlying neural correlate of CRCI in older long-term survivors of cancer.

## Figures and Tables

**Table 1.** Demographic and clinical information

Parameters	C+ N=10	C− N=12	HC N=13	*p*
**Age** years				
Mean (SD)	74.70 (5.44)	76.50 (4.28)	75.54 (6.63)	0.752
Median (Range)	72.5 (68-84)	75.5 (71-86)	75.00 (67-88)	
Race* (N, %)				
White or Caucasian	8 (80)	11 (92)	13 (100)	0.373
Black	1 (10)	1 (8)	.	
Asian, Native Hawaiian	1 (10)	.	.	
Other	.	.	.	
**Highest grade** (N, %)				
High school or less	2 (20)	4 (33)	4 (31)	0.805
College or above	8 (80)	8 (67)	9 (69)	
**AJCC Stage** (N, %)				
DCIS	1 (10)	6 (50)	.	
Stage I	1 (10)	4 (33)	.	
Stage II	8 (80)	2 (17)	.	
**Regimen**				
Non-Trastuzumab Regimen (N, %)	9 (90)			
Trastuzumab Regimen (N, %)	1 (10)			

**Abbreviations:** TP1: time point 1, TP2: time point 2, C+: Chemotherapy group, C−: No-chemotherapy group, HC: Healthy control group, BMI: Body mass index, SD: Standard deviation, AJCC: American Joint Committee on Cancer, DCIS: Ductal carcinoma in situ and N = number of subjects. For all the above comparisons, ANOVA or Fisher tests were used (for continuous or categorical data, respectively). Parameters were significant with threshold at *p* of 0.05.

**Table 2.** Gyrification results

Changes	Size (vertexes)	*p*-value (corrected)	Overlap of atlas region	Brain region (DK40)
**C+ group:**				
TP2>TP1	3119	0.00034	100%	Left Pars opercularis
	11925	0.00034	58%	Right Superior Temporal
			42%	Right Middle Temporal
	11806	0.00025	68%	Right Precuneus
			32%	Right Paracentral
	4661	0.00435	100%	Right Fusiform
TP2<TP1	12086	0.03061	87%	Left Superior Parietal
			13%	Left cuneus
**C− group:**				
TP2<TP1	11450	0.00001	41%	Left Fusiform
			37%	Left Lingual
			22%	Left Isthmus Cingulate
	8600	0.00105	100%	Left Paravaginal
	4351	0.00616	100%	Right Lateral Orbitofrontal
	4198	0.0010	100%	Right Inferior Temporal
	3494	0.00021	100%	Right Caudal Middle frontal
**HC group:**				
TP2<TP1	33739	0.00095	36%	Left Superior Frontal
			28%	Left Postcentral
			22%	Left Precuneus
			10%	Left Paracentral
			4%	Left Caudal Anterior Cingulate
	1064	0.00022	100%	Left Transverse Temporal
	15372	0.00040	77%	Right Superior Frontal
			23%	Right Caudal Middle Frontal
	8150	0.00019	100%	Right Supramarginal

**Abbreviations:** TP1: time point 1, TP2: time point 2, C+: Chemotherapy group, C−: No-chemotherapy group, HC: Healthy control group, DK40: Desikan atlas. Cluster size >10. Results were significant with threshold at *p* of 0.05.

## Data Availability

The datasets generated during the current study are not publicly available due to lack of relevant public database to deposit the data but are available from the corresponding author on reasonable request.
